# Depressor action and vasorelaxation of methylene chloride fraction extracted from *Rubus coreanum*

**DOI:** 10.1186/s40885-014-0006-1

**Published:** 2014-11-25

**Authors:** Byung-Sik Yu, Mee-Sung Choi, Dong-Yoon Lim

**Affiliations:** Department of Anesthesiology and Pain Medicine, School of Medicine, Chosun University, Gwangju, Korea; Department of Leisure and Sport, College of Public Health and Welfare, Dongshin University, Naju, Korea; Department of Pharmacology, School of Medicine, Chosun University, 309 Pilmun-daero, Dong-gu, Gwangju 501-759 Korea

**Keywords:** Rubus coreanum (Bokboonja), Methylene chloride (CH2Cl2) fraction, Vasorelaxation, Depressor action, Adrenergic α1-receptor blockade, Activation of NO synthase

## Abstract

**Introduction:**

The present study was designed to examine whether methylene chloride (CH_2_Cl_2_) fraction extracted from *Rubus coreanum* affects the contractility of the isolated thoracic aortic strips and blood pressure of normotensive rats.

**Methods:**

One of the common carotid arteries or of the femoral arteries was catheterized with a polyethylene tubing. The tubing was connected to a pressure transducer, and pulse of the mean arterial blood pressure was recorded on a biological polygraph continuously.

**Results:**

The CH_2_Cl_2_ fraction (range, 200 to 800 μg/mL) significantly depressed both phenylephrine (PE, 10 μM)- and high K^+^ (56 mM)-induced contractile responses of the isolated thoracic aortic strips in a concentration-dependent fashion. In the simultaneous presence of N^ω^-nitro-L-arginine methyl ester hydrochloride (L-NAME) (an inhibitor of nitric oxide [NO] synthase, 300 μM) and the CH_2_Cl_2_ fraction (400 μg/mL), both PE- and high K^+^-induced contractile responses were recovered to the significant level of the corresponding control response in comparison with inhibition of CH_2_Cl_2_ fraction treatment alone. Moreover, in the simultaneous presence of the CH_2_Cl_2_ fraction after pretreatment with 0.4% CHAPS (3-[(3-cholamidopropyl) dimethylammonio]-1-propane sulfonate), both PE- and high K^+^-induced contractile responses were recovered to the significant level of the corresponding control response compared to the inhibitory response of CH_2_Cl_2_ fraction treatment alone. Also, in anesthetized rats, the CH_2_Cl_2_ fraction (range, 0.3 to 3.0 mg/kg) injected into a femoral vein dose-dependently produced depressor responses. This hypotensive action of the CH_2_Cl_2_ fraction was greatly inhibited after treatment with phentolamine (1 mg/kg), chlorisondamine (1 mg/kg), L-NAME (3 mg/kg/30 min), or sodium nitroprusside (30 μg/kg/30 min). Intravenous infusion of the CH_2_Cl_2_ fraction (range, 1.0 to 10.0 mg/kg/30 min) markedly inhibited norepinephrine-induced pressor responses.

**Discussion:**

Taken together, these results demonstrate that the CH_2_Cl_2_ fraction causes vascular relaxation in the isolated rat thoracic aortic strips as well as hypotensive action in anesthetized rats. These vasorelaxation and hypotension of the CH_2_Cl_2_ fraction seem to be mediated at least by the increased NO production through the activation of NO synthase of the vascular endothelium and the inhibitory adrenergic modulation.

**Electronic supplementary material:**

The online version of this article (doi:10.1186/s40885-014-0006-1) contains supplementary material, which is available to authorized users.

## Introduction

Previously, it has been reported that polyphenol compounds isolated from *Rubus coreanum* MIQUEL (PCRC) inhibit the secretory responses of catecholamines (CA) evoked by the stimulation of cholinergic (both muscarinic and nicotinic) receptors as well as by direct membrane depolarization from the isolated perfused adrenal gland of normotensive rats [1] and spontaneously hypertensive rats [2]. It seems that this inhibitory effect of PCRC is exerted by inhibiting both the Ca^2+^ influx into the rat adrenal medullary chromaffin cells and the uptake of Ca^2+^ into the cytoplasmic calcium store partly through the increased nitric oxide (NO) production due to the activation of NO synthase [1],[2].

Polyphenols also act on other targets involved in the metabolism of mammalian cells, including NO, which by itself regulates hemostasis [3], thrombus development [4], and vascular tone [5],[6]. The properties of NO may therefore explain, at least in part, the beneficial effects of plant polyphenols. Several authors have reported that extracts from grapes and wine induce endothelium-dependent relaxation via enhanced generation and/or increased biological activity of NO leading to the elevation of cyclic guanosine monophosphate (cGMP) levels [7]. The critical step for the activation of NO synthase in endothelial cells is the increase in Ca^2+^ concentration leading to the production of NO and the subsequent endothelium-dependent vasorelaxation [8]. The biological activity of NO can be effectively increased by the scavengers of oxygen free radicals [9].

As aforementioned, there are many reports about the effects of red wine on the cardiovascular system. Despite these studies, there are so far few reports on *in vitro* functional effects of fractions isolated from Bokboonja wine on the cardiovascular system. Therefore, the aim of the present study was to investigate the effects of some fractions isolated from Bokboonja wine on blood pressure and on the contractility of the isolated rat thoracic aorta and to clarify their mechanism of action in order to supply information for isolation of active antihypertensive components.

## Methods

### Experimental procedure

All procedures involving animal experiment were approved by the Committee of Experimental Animals, Chosun University School of Medicine.

#### Vasorelaxation

Mature male Sprague-Dawley rats (purchased from DAMOOL SCIENCE; International Customer Service, Seoul, Korea), weighing 200 to 300 g, were used in the experiment. The animals were housed individually in separate cages, and food (Cheil Animal Chow, Seoul, Korea) and tap water were allowed *ad libitum* for at least a week to adapt to experimental circumstances. On the day of the experiment, a rat was anesthetized with thiopental sodium (50 mg/kg) intraperitoneally and tied in supine position on a fixing panel.

1. Isolation of thoracic aortic strips: The thorax was opened by a midline incision, and the heart and surrounding area were exposed by placing three hook retractors. The heart and a portion of the lung were not removed but pushed over to the right side and covered by saline-soaked gauze pads in order to obtain enough working space for isolating the thoracic aortic vessel. The aorta was isolated from the proximal part of the heart to the vicinity of the liver and immediately immersed in cold Krebs solution. The blood within the aorta was rapidly removed. The aorta was cut into the ring of 4-to 5-mm length.
2. Recording of mechanical activity: The ring segment of the aorta was mounted in a muscle bath by sliding the ring over two parallel stainless steel hooks (0.15 mm in diameter). The lower hook was fixed at the bottom of the bath and the upper was connected to an isometric transducer (Grass FT. 03). The signal from the transducer was displayed on a polygraph (Grass Instruments Model 79; Grass Instrument Co., Quincy, MA, USA). The volume of the bath was 25 mL and the bath solution was saturated with 95% O_2_ and 5% CO_2_ at 37°C. The composition (mM) of Krebs was as follows: NaCl, 118.4; KCl, 4.7; CaCl_2_, 2.5; MgCl_2_, 1.18; NaHCO_3_, 25; KH_2_PO_4_, 1.2; and glucose, 11.7. The final pH of the solution was maintained at 7.4 to 7.5. During equilibration period of 2 h, the resting tension was adjusted to 0.5 g. After the equilibration period, the ring was challenged with 35 mM KCl twice, and if it responded with contraction, the proper experiment was started. Vasoconstrictors were administered into the bath in order to obtain dose-response curves. In the subsequent experiments, under the presence of extracts of *Rubus coreanum*, some vasoconstrictors were administered, respectively. The data were expressed as percentage of the control tension.
3. Removal of endothelium: A solution containing 0.4% 3-[(3-cholamidopropyl) dimethylammonio]-1-propane sulfonate (CHAPS) was perfused for 30 s to remove the endothelium [10], followed by washout with a drug-free solution. The effect of CHAPS was confirmed by the absence of a flow increase due to 10^−6^ M acetylcholine and the presence of a response to 10^−6^ M sodium nitroprusside before the experiments were started. The vasoconstrictor-induced response of non-treated (control) and CHAPS-treated preparations was compared in parallel.

#### Blood pressure

1. Preparation for measurement of arterial pressure: The animal was tied in supine position on a fixing panel to insert a T-formed cannula into the trachea for securing free air passage. The rectal temperature was maintained at 37°C to 38°C by a thermostatically controlling blanket and heating lamp throughout the course of the experiment.
2. Measurement of blood pressure: In order to observe the change of arterial pressure, one of the common carotid arteries or of the femoral arteries was catheterized with a polyethylene tubing (outside diameter [od], 0.5 mm). The tubing was connected to a pressure transducer (Gould Co., Quincy, MA, USA), and pulse of the mean arterial blood pressure was recorded on a biological polygraph continuously (Grass Co.). The chart speed was adjusted to 2 cm/min. The artery tubing was filled with heparin solution (400 IU) to prevent the blood coagulation during the experiment. Another cannulation with a polyethylene tubing (od, 0.3 mm) was made into a femoral vein for the administration of drugs and supplemental anesthetic agents as needed to maintain light surgical anesthesia. Each rat was left undisturbed for at least 30 min after completion of the operative procedures to permit cardiovascular parameters to be stabilized, and drugs under investigation were administered at intervals of 60 min.

#### Fractionation of *Rubus coreanum*

**Figure 1:**
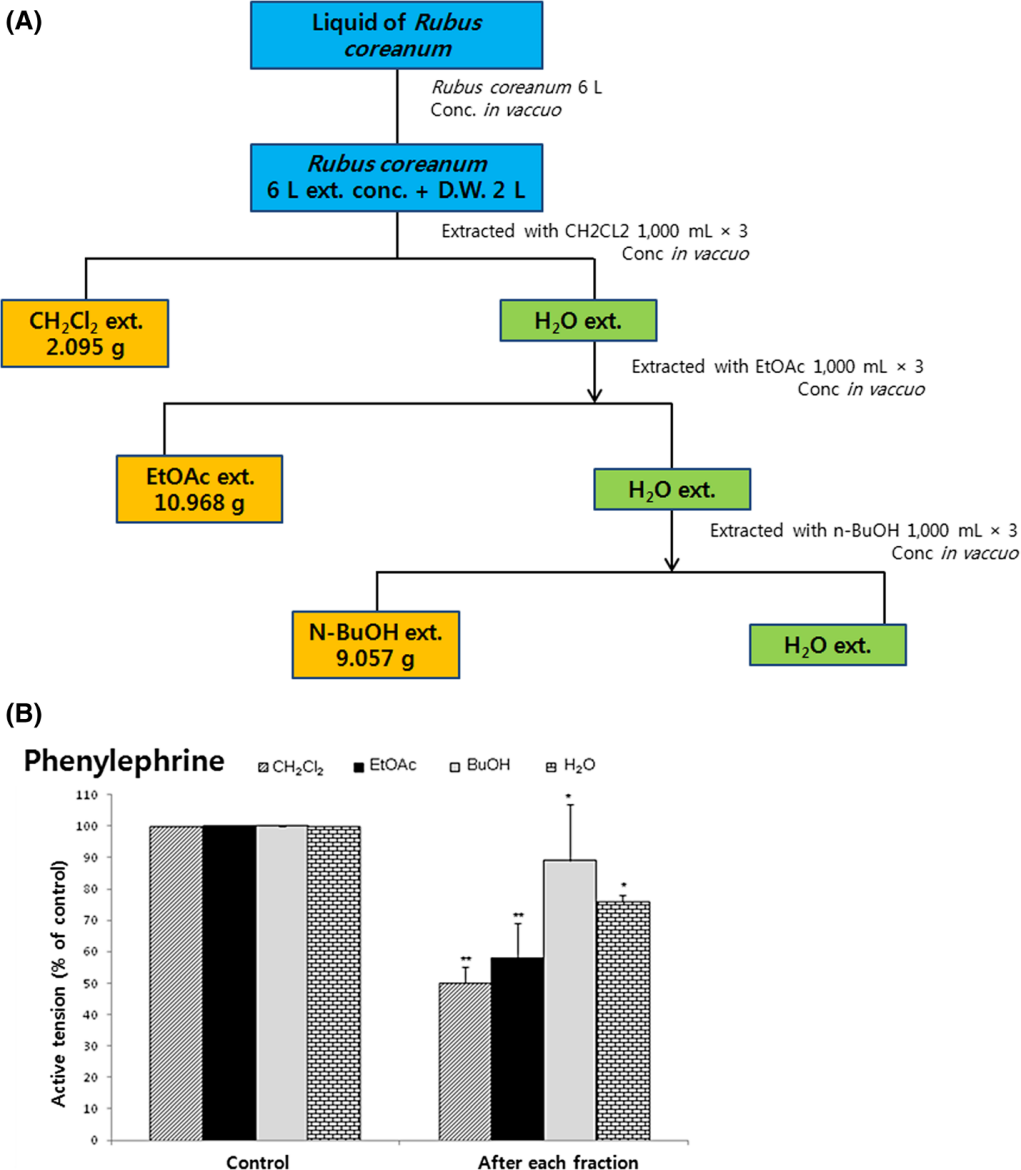
**(A) Fractionation procedure of** ***Rubus coreanum*** **and (B) comparative effects of four fractions (water [H**_**2**_**O], butanol [BuOH], ethylacetate [EtOAc], and methylene chloride [CH**_**2**_**Cl**_**2**_**]) extracted from** ***Rubus coreanum*** **on the inhibition of phenylephrine (PE)-induced contractile responses in the isolated thoracic aortic strips of rats.** The contractile responses were induced by adding 10 μM PE at a 120-min interval after adaptation with normal Krebs solution for 2 h prior to initiation of the experimental protocol. Each *column* denotes active tension induced evoked by 10 μM PE before and after adding fractions (400 μg/mL) of EtOAc, CH_2_Cl_2_, BuOH, and H_2_O, respectively. *Vertical bars* represent the standard error of the mean. *Ordinate*: the active tension (% of control, 1.6 ± 0.1 g [10 μM]). *Abscissa*: after treatment of each fraction. Statistical difference was obtained by comparing its control with each fraction-pretreated group. *p < 0.05. **p < 0.01.

Fractionation of *Rubus coreanum* extract was made from a 1-year-old wine brewed from *Rubus coreanum* MIQUEL at the Research Institute of Bokboonja, Gochang County, Cheollabukdo Province, Korea, as shown in Figure 1A: wine of *Rubus coreanum* was concentrated in a vacuum. And then, it was extracted with methylene chloride (CH_2_Cl_2_) followed by extraction with ethylacetate (EtOAc) and N-butanol. These fractions were concentrated by vacuum, evaporated and atomized, and lyophilized by freeze dryer (Coldvac-80; Hanil R&D, Seoul, Korea). Extract of 2.095 g CH_2_Cl_2_, 10.968 g EtOAc, and 9.057 g N-butanol was obtained from 6 L Bokboonja wine, respectively. The working solution of these extracts was prepared by dissolving in 0.9% NaCl solution or dimethyl sulfoxide (DMSO) on the day of each experiment and filtered before administration and diluted appropriately with Krebs-bicarbonate solution (final concentration of alcohol was less than 0.1%).

### Statistical analysis

The statistical difference between the control and the pretreated groups was determined by the Student *t* test and analysis of variance test. A *p* value of less than 0.05 was considered to represent statistically significant changes unless specifically noted in the text. Values given in the text refer to means and the standard errors of the mean. The statistical analysis of the experimental results was made by a computer program described by Tallarida and Murray [11].

### Drugs and their sources

The following drugs were used: phenylephrine (PE) hydrochloride, potassium chloride, N^ω^-nitro-L-arginine methyl ester hydrochloride (L-NAME), CHAPS, norepinephrine bitartrate (Sigma-Aldrich Chemical Co., St. Louis, MO, USA), chlorisondamine chloride, phentolamine mesylate (Ciba Pharmaceutical Co., Summit, NJ, USA), thiopental sodium, and heparin sodium (Daehan Choongwae Pharm. Co., Seoul, Korea). Drugs were dissolved in distilled water (stock) and added to the normal Krebs solution as required except the CH_2_Cl_2_ fraction, which were dissolved in DMSO and diluted appropriately with Krebs-bicarbonate solution (final concentration of DMSO was less than 0.1%). Concentrations of all drugs except the CH_2_Cl_2_ fraction used were expressed in terms of molar base.

## Results

### Effects of four fractions extracted from *Rubus coreanum*on PE-induced contractile responses in the thoracic aortic strips of normotensive rats

The effects of four fractions extracted from *Rubus coreanum* on PE-induced contractile responses in the rat aorta with an intact endothelium were examined. In the present study, the CH_2_Cl_2_ fraction itself did not produce any effect on the resting tension in the aortic strips with an intact endothelium isolated from rats (data not shown). In a previous study, it has been found that PCRC causes vascular relaxation in the isolated aortic strips of spontaneously hypertensive rats (SHRs) at least partly by the increased NO production through the activation of NO synthase of the vascular endothelium, but not through the activation of cyclooxygenase [12]. Therefore, it was attempted to examine the effects of four fractions (EtOAc, CH_2_Cl_2_, N-butanol [BuOH], and water [H_2_O]) isolated from *Rubus coreanum* M. on PE-induced contractile responses in the isolated rat aortic strips. As shown in Figure 1B, in the presence of the CH_2_Cl_2_ fraction (400 μg/mL), EtOAc fraction (400 μg/mL), BuOH fraction (400 μg/mL), and H_2_O fraction (400 μg/mL) 5 min before addition of PE, the contractile responses of PE (10^−5^ M) were significantly reduced to 50% ± 1% (*p* < 0.01, *n* = 6), 58% ± 11% (*p* < 0.01, *n* = 6), 90% ± 18% (*p* < 0.01, *n* = 8), and 78% ± 2% (*p* < 0.05, *n* = 6) of the corresponding control response (1.6 ± 0.1 g), respectively. Based on these results, for the PE-induced contractile response, the following rank order of inhibitory potency was obtained: CH_2_Cl_2_ fraction > EtOAc fraction > H_2_O fraction > BuOH fraction. Therefore, in all subsequent experiments, the CH_2_Cl_2_ fraction (400 μg/mL) only was used.

### Effects of the CH_2_Cl_2_ fraction isolated from *Rubus coreanum* on contractile responses induced by PE and high K^+^in the thoracic aortic strips of normotensive rats

**Figure 2:**
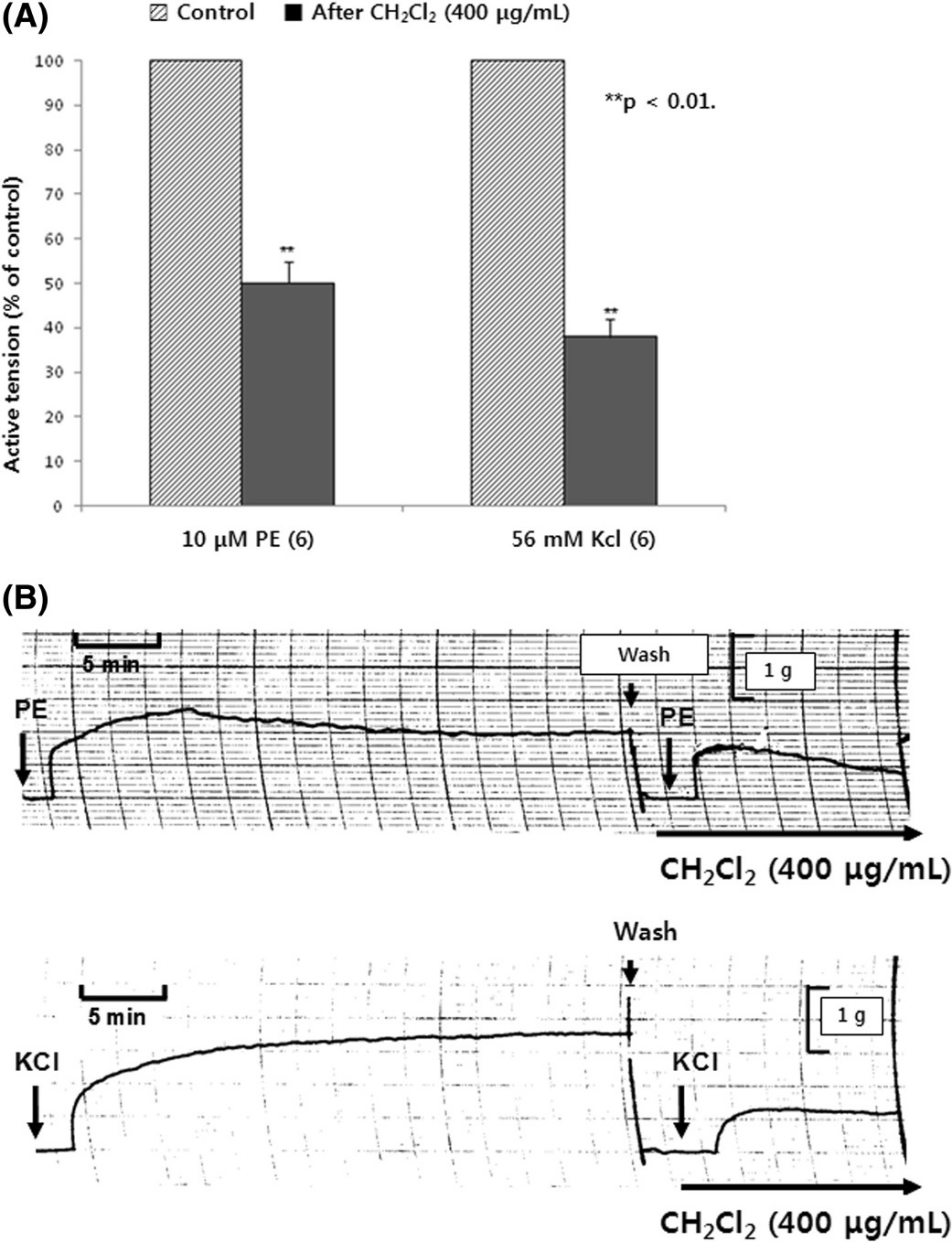
**(A) Influence of methylene chloride (CH**_**2**_**Cl**_**2**_**) fraction on phenylephrine (PE)- and high potassium (KCl)-induced contractile responses and (B) the typical tracing showing the effect of CH**_**2**_**Cl**_**2**_**fraction on PE- and high potassium (KCl)-induced contractile response in the isolated rat aortic strips.** *Black column* and *brick column* denote active tension induced evoked by 10 μM PE before and after adding 400 μg/mL of CH_2_Cl_2_ fraction, respectively. Other methods are the same as in Figure 1. **p < 0.01. **(A)** *Left*: PE-induced contractile response (control); *right*: PE-induced contractile response in the presence of CH_2_Cl_2_ fraction (400 μg/mL). **(B)** *Left*: KCl-induced contractile response (control); *right*: KCl-induced contractile response in the presence of CH_2_Cl_2_ fraction (400 μg/mL). At *arrow marks*, the indicated doses of PE (10 μM) and KCl (56 mM) were added into the bath, respectively. The chart speed was 5 mm/min.

PE is a selective agonist of adrenergic α_1_-receptors, which exhibits vasoconstriction. To establish the inhibitory effect of the CH_2_Cl_2_ fraction on PE (10^−5^ M)-induced contractile responses, in the presence of the CH_2_Cl_2_ fraction at 400 μg/mL 5 min before addition of PE, the contractile response of PE (10^−5^ M) was greatly reduced to 50% ± 5% (*p* < 0.01, *n* = 6) of the corresponding control response (1.8 ± 0.2 g) (Figure 2).

High K^+^ exerts two distinct effects on cells: (1) depolarization of the cell membrane and (2) depolarization-induced influx of calcium via voltage-dependent calcium channels [13]. When added to the bath, high potassium at 5.6 × 10^−2^ M, which is a membrane-depolarizing agent, caused an increase in aortic contraction (2.4 ± 0.2 g). As shown in Figure 2, high potassium (5.6 × 10^−2^ M)-induced contractile response after preloading with 400 μg/mL of the CH_2_Cl_2_ fraction 5 min before high potassium was significantly reduced to 62% ± 8% (*p* < 0.01, *n* = 6) of the corresponding control response (2.4 ± 0.2 g).

### Influence of the CH_2_Cl_2_fraction plus L-NAME on the contractile responses evoked by PE and high potassium in the thoracic aortic strips of normotensive rats

In a previous study, it has been demonstrated that PCRC inhibits the CA secretion evoked by cholinergic stimulation and direct membrane depolarization from the perfused rat adrenal medulla, which was blocked in the presence of L-NAME, a NO synthase inhibitor [2]. These results suggest that PCRC can inhibit the CA release at least partly through the activation of NO synthase in the rat adrenal medulla. Therefore, in the presence of L-NAME, it was likely interesting to compare the effects of the CH_2_Cl_2_ fraction on the contractile responses induced by high potassium and PE.

**Figure 3:**
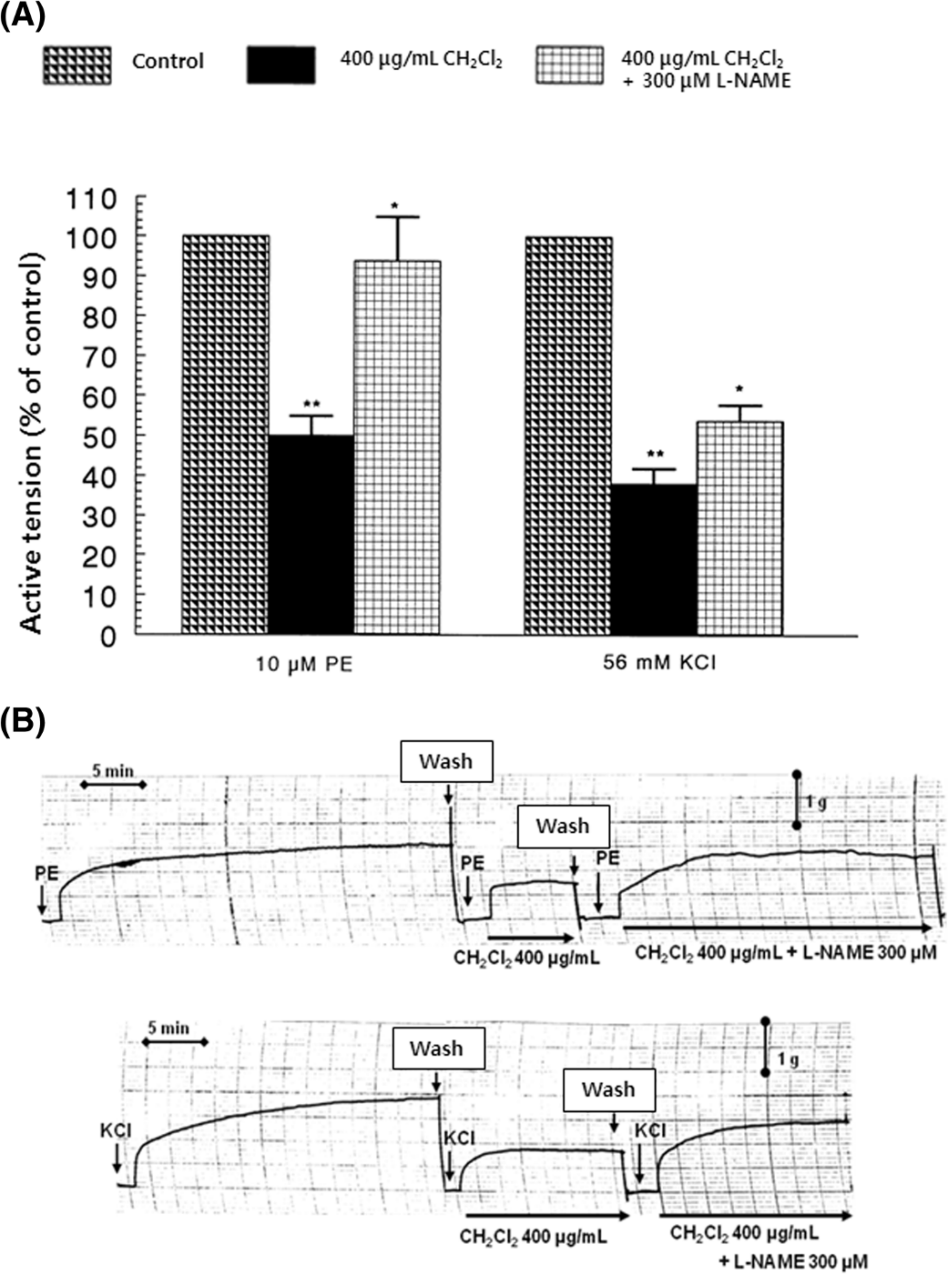
**(A) Influence of methylene chloride (CH**_**2**_**Cl**_**2**_**) fraction plus N** ^**ω**^**-nitro-L-arginine methyl ester hydrochloride (L-NAME) on the contractile responses evoked by phenylephrine (PE) and high potassium (KCl) and (B) the typical tracing showing the effect of CH**_**2**_**Cl**_**2**_**fraction plus L-NAME on (** ***upper panel*** **) PE- and (** ***lower panel*** **) high potassium-induced contractile response in the isolated rat aortic strips.** Statistical difference was obtained by comparing the control with the CH_2_Cl_2_ fraction-pretreated group or CH_2_Cl_2_ fraction (400 μg/mL) plus L-NAME (300 μM). Other methods are the same as in Figure 2. *p < 0.05. **p < 0.01. **(A)** *Left*: PE-induced contractile response (control); *middle*: PE-induced contractile response in the presence of CH_2_Cl_2_ fraction (400 μg/mL); *right*: PE-induced contractile response in the presence of CH_2_Cl_2_ fraction (400 μg/mL) plus L-NAME (300 μM). **(B)** *Left*: KCl-induced contractile response (control); *middle*: KCl-induced contractile response in the presence of CH_2_Cl_2_ fraction (400 μg/mL); *right*: KCl-induced contractile response in the presence of CH_2_Cl_2_ fraction (400 μg/mL) plus L-NAME (300 μM).

In the simultaneous presence of CH_2_Cl_2_ fraction (400 μg/mL) and L-NAME (300 μM), the aortic contractile response evoked by PE (10^−5^ M) was recovered to 94% ± 11% (*p* < 0.05, *n* = 10) of the control in comparison with the inhibitory response of CH_2_Cl_2_ fraction treatment alone (50% ± 5%) from the resting tension level as shown in Figure 3. High potassium (5.6 × 10^−2^ M)-induced contractile response in the simultaneous presence of CH_2_Cl_2_ fraction (400 μg/mL) and L-NAME (300 μM) was recovered to 54% ± 4% (*p* < 0.05, *n* = 6) of the corresponding control compared with the inhibitory response of CH_2_Cl_2_ fraction treatment alone (38% ± 4%) from the resting tension level (Figure 3).

### Influence of the CH_2_Cl_2_fraction plus CHAPS on contractile responses induced by PE and high potassium in the thoracic aortic strips of normotensive rats

As shown in Figure 3, CH_2_Cl_2_ fraction-induced vasorelaxation was markedly blocked in the presence of L-NAME, a NO synthase inhibitor. Therefore, it is likely interesting to examine the effects of CHAPS, a detergent which suppresses endothelial function [10], on CH_2_Cl_2_ fraction-induced inhibitory responses to the contractile active tension evoked by high potassium and PE.

**Figure 4:**
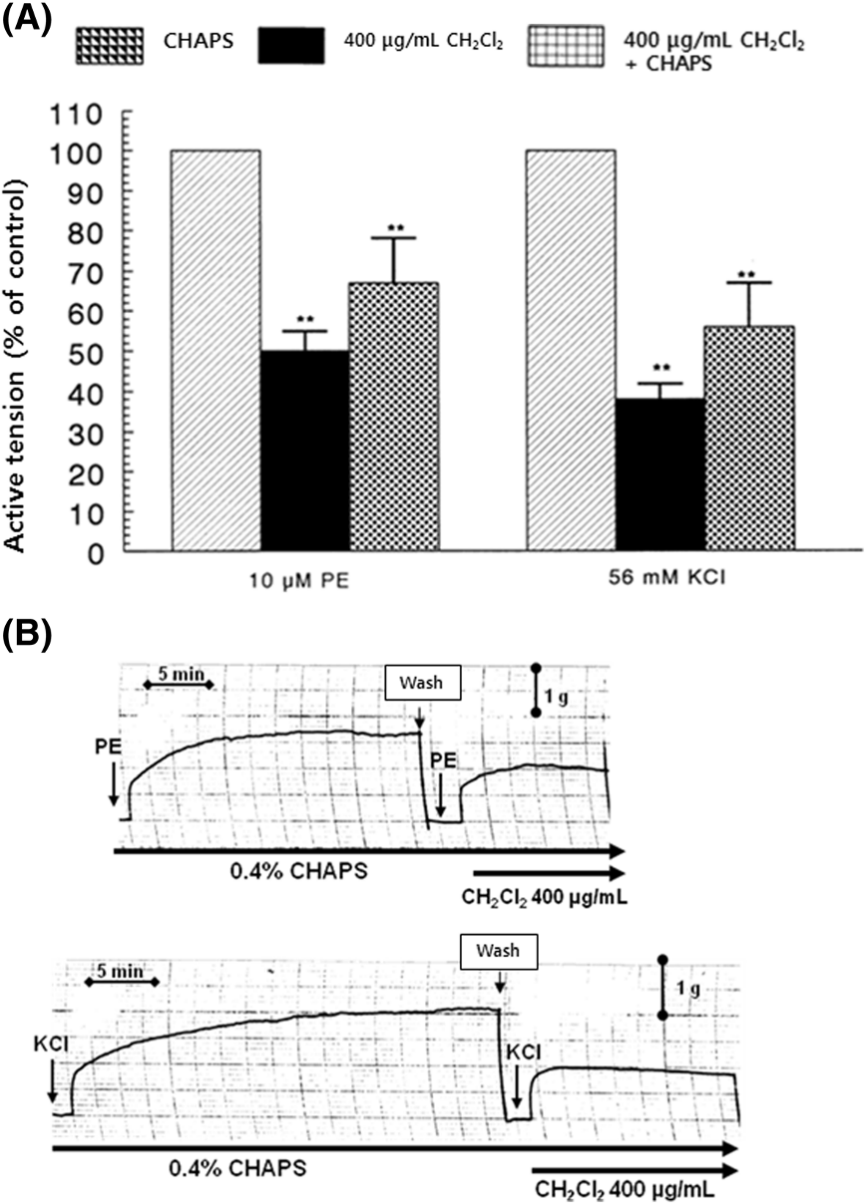
**(A) Influence of 3-[(3-cholamidopropyl) dimethylammonio]-1-propane sulfonate (CHAPS) plus methylene chloride (CH**_**2**_**Cl**_**2**_**) fraction on contractile responses induced by phenylephrine (PE) and high potassium (KCl) and (B) the representative tracing of CHAPS plus CH**_**2**_**Cl**_**2**_**fraction effect on contractile responses induced by PE and high potassium in the isolated rat aortic strips.** Other methods are the same as in Figure 3. **p < 0.01. At *arrow marks*, PE (10 μM) and KCl (56 mM) were added into CHAPS-pretreated aortic strips. **(A)** PE-induced contractile response after CH_2_Cl_2_ fraction (400 μg/mL) treatment in a CHAPS-pretreated aortic strip. **(B)** High KCl-induced contractile response after CH_2_Cl_2_ fraction (400 μg/mL) treatment in a CHAPS-pretreated aortic strip. The chart speed was 5 mm/min.

In the presence of the CH_2_Cl_2_ fraction (400 μg/mL) after pretreatment with 0.4% CHAPS, the aortic contractile response evoked by PE (10^−5^ M) was recovered to 67% ± 13% (*p* < 0.01, *n* = 6) of the corresponding control compared with the inhibitory response of CH_2_Cl_2_ fraction treatment alone (38% ± 4%) from the resting tension level, as shown in Figure 4. High potassium (5.6 × 10^−2^ M)-induced contractile response in the simultaneous presence of the CH_2_Cl_2_ fraction (400 μg/mL) after pretreatment with CHAPS was recovered to 67% ± 13% (*p* < 0.01, *n* = 6) of the corresponding control compared with the inhibitory response of CH_2_Cl_2_ fraction treatment alone (38% ± 4%) from the resting tension level (Figure 4).

### Effects of intravenous CH_2_Cl_2_fraction on blood pressure in the anesthetized normotensive rats

**Figure 5:**
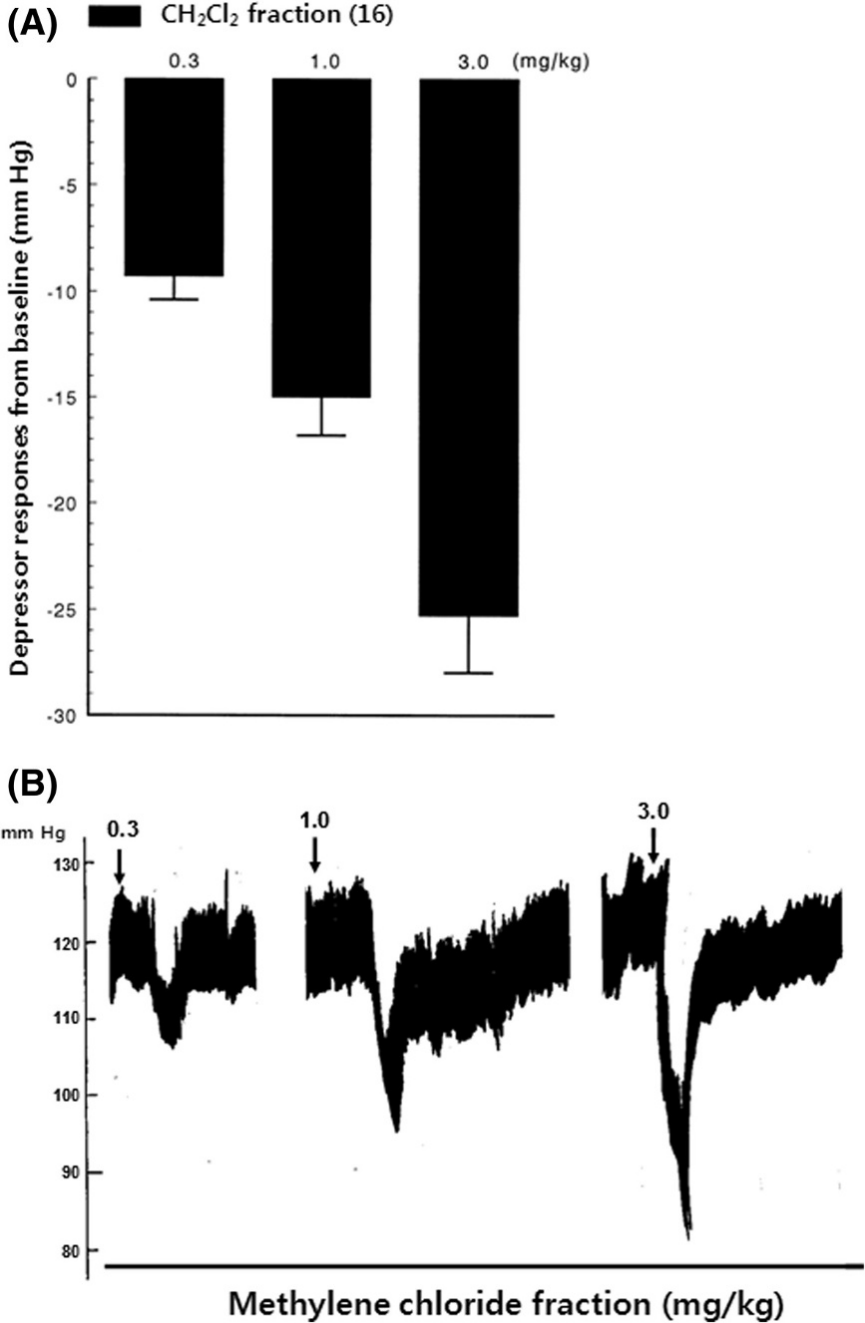
**(A) Dose-dependent hypotensive effects of methylene chloride (CH**_**2**_**Cl**_**2**_**) fraction and (B) the typical tracings of CH**_**2**_**Cl**_**2**_**fraction-induced hypotensive action in an anesthetized rat.** CH_2_Cl_2_ fraction (0.3, 1.0, and 3.0 mg/kg, respectively) was administered into a femoral vein. Arterial blood pressure from preinjection level was expressed in millimeters of mercury. CH_2_Cl_2_ fraction at the indicated doses (0.3, 1.0, and 3.0 mg/kg) was injected intravenously at the *arrow marks.*

All rats used in this study were allowed to be stabilized at least for 60 min before experimental protocols were initiated. When cardiovascular parameters were stabilized, the CH_2_Cl_2_ fraction (range, 0.3 to 3.0 mg/kg) was given into a femoral vein of the normotensive rats anesthetized with thiopental sodium and urethane. The CH_2_Cl_2_ fraction produced a dose-related and potent fall in arterial blood pressure. However, an equivalent volume of 0.9% saline given into a femoral vein did not produce any changes in blood pressure of the normotensive rats. As shown in Figure 5, intravenous 0.3 mg of CH_2_Cl_2_ fraction induced a fall in mean arterial pressure by 9.1 ± 1.1 mm Hg from the original baseline of 122.2 ± 4.0 mm Hg, but increasing doses of the CH_2_Cl_2_ fraction to 1.0 and 3.0 mg/kg intravenously (i.v.) showed decreased mean arterial pressures of 15.0 ± 1.8 and 25.3 ± 2.7 mm Hg, respectively, from the preinjection level of the baseline from ten rats. All of the above experimental results were statistically significant from the corresponding preinjection values (*p* < 0.01).

### Influence of phentolamine, chlorisondamine, L-NAME, and sodium nitroprusside on the CH_2_Cl_2_fraction-induced depressor action

**Figure 6:**
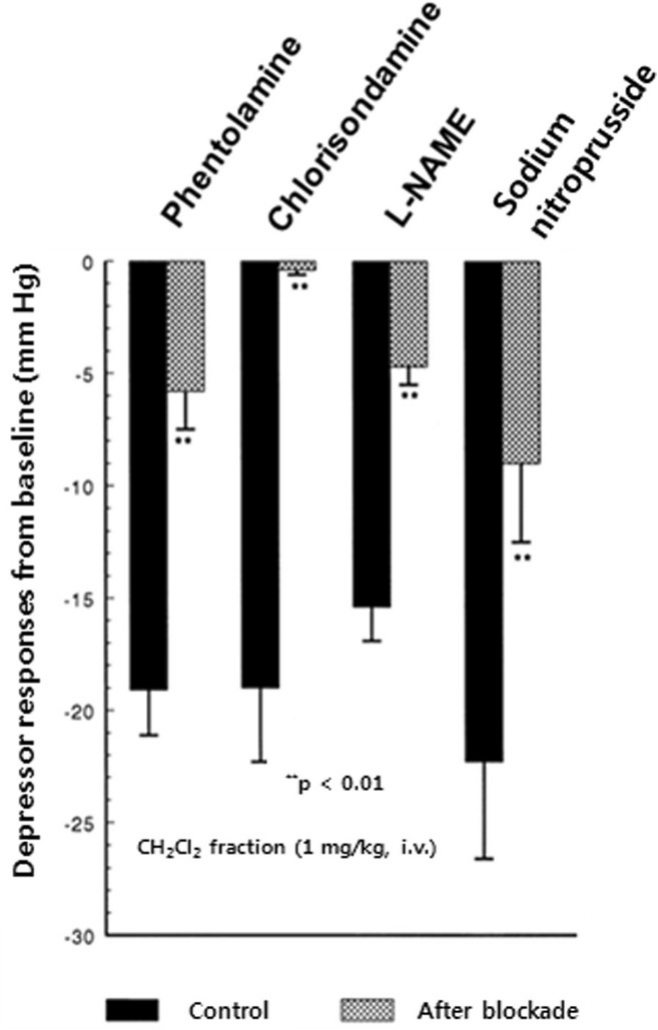
**Influence of phentolamine, chlorisondamine, 3-[(3-cholamidopropyl) dimethylammonio]-1-propane sulfonate (L-NAME), and nitroprusside on methylene chloride (CH**_**2**_**Cl**_**2**_**) fraction-induced hypotensive action in the anesthetized rats.** Phentolamine (1 mg/kg), chlorisondamine (1 mg/kg), L-NAME (3 mg/kg/30 min), and sodium nitroprusside (30 μg/kg/30 min) were given intravenously (i.v.), respectively, after obtaining CH_2_Cl_2_ fraction-induced hypotensive action. Statistical difference was analyzed by comparing control response with that after treatment with each blockade. **p < 0.01.

**Figure 7:**
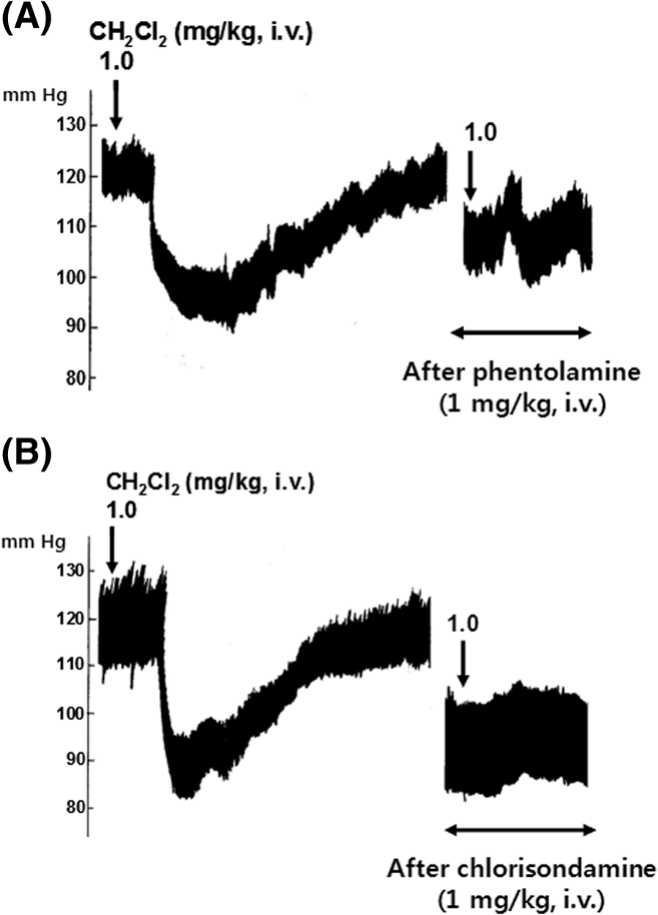
**A typical tracing showing the effects of (A) phentolamine and (B) chlorisondamine on the hypotensive action of methylene chloride (CH**_**2**_**Cl**_**2**_**) fraction in the anesthetized rats.** Phentolamine (1 mg/kg) and chlorisondamine (1 mg/kg) were given intravenously after obtaining control hypotensive responses of CH2Cl2 fraction, respectively. *i.v.*, intravenously.

**Figure 8:**
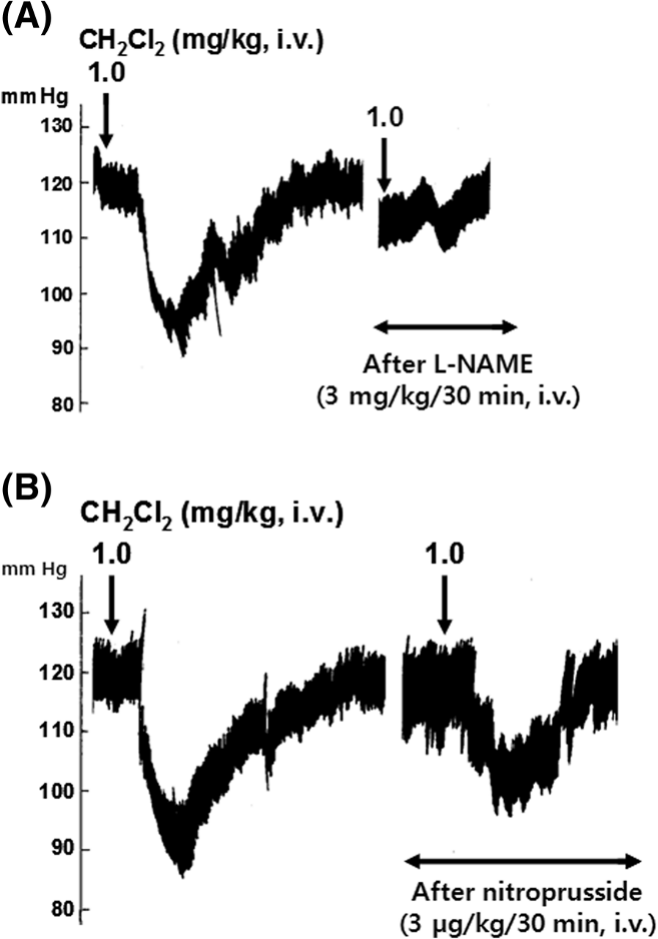
**A typical tracing showing the effects of (A) N** ^**ω**^**-nitro-L-arginine methyl ester hydrochloride (L-NAME) and (B) nitroprusside on the hypotensive action of methylene chloride (CH**_**2**_**Cl**_**2**_**) fraction in the anesthetized rats.** L-NAME (3 mg/kg/30 min) and nitroprusside (30 μg/kg/30 min) were given intravenously after obtaining control hypotensive responses of CH_2_Cl_2_ fraction, respectively. *i.v.*, intravenously.

In eight rats, in order to examine the relationship between adrenergic α-receptors and CH_2_Cl_2_ fraction-induced depressor action, phentolamine (1.0 mg/kg) was given i.v. after obtaining the control responses of the intravenous CH_2_Cl_2_ fraction. In the presence of a phentolamine effect, depressor response induced by the intravenous CH_2_Cl_2_ fraction (1.0 mg/kg) was greatly depressed to −5.8 ± 1.7 mm Hg (*p* < 0.01) from the preinjection level of the baseline as compared with the control depressor response (−19.1 ± 2.9 mm Hg) as shown in Figures 6 and 7A. Chlorisondamine (1.0 mg/kg), an autonomic ganglionic blocking agent, was given slowly into a femoral vein. Following the administration of chlorisondamine, the baseline of blood pressure was reduced from 119.2 ± 4.1 to 70.2 ± 3.9 mm Hg. In ten rats, intravenous CH_2_Cl_2_ fraction (1.0 mg/kg)-induced depressor response after chlorisondamine treatment was markedly inhibited by −0.4 ± 0.2 mm Hg (*p* < 0.01) as compared with the control depressor response (−19.0 ± 3.3 mm Hg), as shown in Figures 6 and 7B. Intravenous infusion of L-NAME (3 mg/kg/30 min), an inhibitor of NO synthase, into a femoral vein resulted in a significant decrease by −4.7 ± 0.8 mm Hg (*p* < 0.01, *n* = 20) in the blood pressure as compared with the control depressor response (−15.4 ± 1.5 mm Hg), as shown in Figures 6 and 8A. In six rats, in order to examine the relationship between NO and CH_2_Cl_2_ fraction-induced depressor action, sodium nitroprusside (30 μg/kg/30 min) was infused i.v. after obtaining the control responses of the intravenous CH_2_Cl_2_ fraction. In the presence of a sodium nitroprusside effect, depressor response induced by the intravenous CH_2_Cl_2_ fraction (1.0 mg/kg) was greatly depressed to −9.3 ± 3.5 mm Hg (*p* < 0.01) as compared with the control depressor response (−22.3 ± 4.3 mm Hg) as shown in Figures 6 and 8B.

### Influence of the intravenous CH_2_Cl_2_fraction on norepinephrine-evoked pressor responses in the anesthetized rats

As shown in Figures 2, 6, and 7, the CH_2_Cl_2_ fraction greatly inhibited PE-induced contractile response of the aortic strip of normotensive rats, and also, CH_2_Cl_2_ fraction-induced depressor responses were significantly reduced by phentolamine and chlorisondamine; this suggests that the CH_2_Cl_2_ fraction might cause hypotension through the blockade of peripheral adrenergic α-receptors. It is also of interest to examine the effect of the CH_2_Cl_2_ fraction on norepinephrine-evoked pressor responses. When cardiovascular parameters were stabilized for 30 min before the experimental protocols were initiated, the administration of physiological saline solution in a volume of 0.2 mL into a femoral vein did not cause any changes in arterial blood pressure. Then, it was tried to test the effect of the CH_2_Cl_2_ fraction on norepinephrine-induced hypertensive responses in the anesthetized rats.

**Figure 9:**
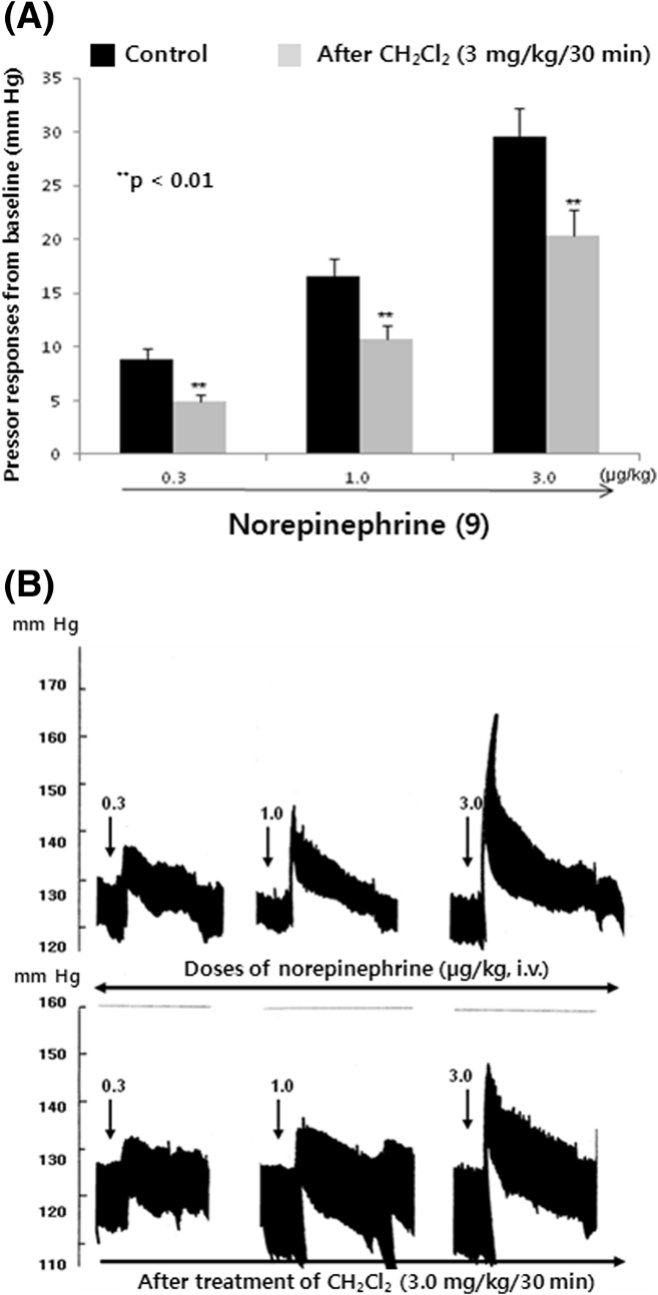
**(A) Influence of intravenous methylene chloride (CH**_**2**_**Cl**_**2**_**) fraction on norepinephrine (NE)-evoked pressor responses and (B) the representative tracing of effect of CH**_**2**_**Cl**_**2**_**fraction on intravenous NE-induced pressor responses in anesthetized rats.** CH2Cl2 fraction (3.0 mg/kg/30 min) was given intravenously after obtaining the corresponding control responses of intravenous NE (0.3, 1.0, and 3.0 μg/kg, respectively). **p < 0.01. At *arrow marks*, the indicated doses (0.3, 1.0, and 3.0 μg/kg) of NE were administered into a femoral vein. **(A)** NE-induced hypertensive responses in a non-treated rat. **(B)** NE-induced hypertensive responses in a CH_2_Cl_2_ fraction-pretreated rat. CH_2_Cl_2_ fraction was infused into a femoral vein with a rate of 3 mg/kg/30 min. Arterial blood pressure from preinjection level was expressed in -mmHg. The chart speed was 10 mm/min. *i.v.*, intravenously.

In nine rats, as shown in Figure 9, norepinephrine at doses of 0.3, 1.0, and 3.0 μg/kg i.v. caused dose-dependent pressor responses of 8.9 ± 0.8, 16.5 ± 1.7, and 29.6 ± 2.6 mm Hg from the original baseline (122.1 ± 5.0 mm Hg), respectively. After infusion of the CH_2_Cl_2_ fraction with a rate of 3.0 mg/kg/30 min, hypertensive responses of norepinephrine at doses of 0.3, 1.0, and 3.0 μg/kg were inhibited maximally to 4.9 ± 0.6 mm Hg (*p* < 0.01), 10.8 ± 1.2 mm Hg (*p* < 0.01), and 20.3 ± 2.5 mm Hg (*p* < 0.01) of control responses at the above same doses, respectively.

## Discussion

The present experimental results demonstrate that the CH_2_Cl_2_ fraction causes depressor action as well as vasorelaxation in the isolated aortic strips of normotensive rats at least partly by the increased NO production through the activation of NO synthase of the vascular endothelium.

In support of this idea, it has been demonstrated that PCRC inhibits the CA secretory responses evoked by stimulation of cholinergic (both muscarinic and nicotinic) receptors as well as by direct membrane depolarization from the isolated perfused adrenal gland of the normotensive rats [1] and SHRs [14]. It seems that this inhibitory effect of PCRC is exerted by inhibiting both the Ca^2+^ influx into the rat adrenal medullary chromaffin cells and the uptake of Ca^2+^ into the cytoplasmic calcium store partly through the increased NO production due to the activation of NO synthase [1],[14]. In the present study, the CH_2_Cl_2_ fraction elicited a concentration-dependent inhibition in phenylephrine-induced contractile responses of rat aortic rings with a functional endothelium. This effect was greatly abolished in the absence of a functional endothelium by treatment with CHAPS, which is a detergent for the removal of endothelium, indicating that the vasodilator effect of the CH_2_Cl_2_ fraction is dependent on endothelium-derived relaxing factors. To evaluate the participation of NO in the vasorelaxant activity of the CH_2_Cl_2_ fraction, aortic rings were also treated with L-NAME, a classical NO synthase inhibitor. In the present experimental condition, the CH_2_Cl_2_ fraction-induced vasodilatation was markedly blocked, as similarly observed in endothelium-denuded aortic rings by CHAPS, suggesting that NO is the main endothelium-derived relaxing factor involved in CH_2_Cl_2_ fraction activity. The present results are fully in accordance with previous findings obtained from red wines and grapes. Previously, it has been reported that red wines and grapes exhibit endothelium-dependent relaxation of blood vessels via enhanced generation and/or increased biological activity of NO, leading to the elevation of cGMP levels [6],[7],[15],[16]. *In vivo*, the polyphenol compounds of red wine (PCRW) were shown to reduce blood pressure in normotensive and hypertensive rats [17]-[19]. In denuded aortic rings, a PCRW concentration 103-fold higher was necessary to induce relaxation [20],[21]. Besides NO, red wine affected the formation of other mediators of vascular tone, such as endothelium-derived hyperpolarizing factor [20] and prostacyclin [22]. The mechanisms underlining NO-dependent vasorelaxation caused by PCRW were investigated [6],[8],[23]. In addition to the increased NO synthase activity, PCRW may prolong the half-life and increase the bioavailability of NO, by reducing its degradation mediated by reactive oxygen species [24]. It has also been shown that Provinol elicited endothelium-dependent relaxation of rat femoral artery by the Ca^2+^-induced increase of NO synthase activity and by protecting NO from degradation [6]. Yu et al. [2] have found that PCRW inhibits the CA secretory responses evoked by stimulation of cholinergic (both muscarinic and nicotinic) receptors as well as by direct membrane depolarization from the isolated perfused adrenal gland of the normotensive rats. It seems that this inhibitory effect of PCRW is mediated by blocking the influx of both ions through Na^+^ and Ca^2+^ channels into the rat adrenomedullary chromaffin cells as well as by inhibiting the release of Ca^2+^ from the cytoplasmic calcium store, which are due at least partly to the increased NO production through the activation of NO synthase.

Generally, endothelium-derived NO plays an important role in the control of vascular homeostasis. NO modulates vascular tone and growth of vascular smooth muscle cells and decreases platelet adhesion and aggregation. It also decreases the adherence of other blood components [5],[25]. A decrease in NO production or bioavailability is closely associated with endothelial dysfunction or injury, which is an important factor in pathologies such as atherosclerosis, restenosis, and hypertension [26]. PCRW and a grape skin extract also reduced blood pressure in males in several models of experimental hypertension [19],[27]-[30], which was related to a combination of vasodilator and antioxidant actions. Pechanova and his colleagues [27] also provided evidence that Provinol partially prevents L-NAME-induced hypertension, cardiovascular remodeling, and vascular dysfunction via the increase of NO synthase activity and prevention of oxidative stress. In the present study, the intravenous CH_2_Cl_2_ fraction-induced hypotensive response was significantly inhibited by pretreatment with L-NAME or sodium nitroprusside. In light of these results, it seems that the CH_2_Cl_2_ fraction may produce hypotensive action at least through the increased NO production by eNOS activation. Thus, in view of the beneficial effects of plant polyphenols, the present results of the CH_2_Cl_2_ fraction should shed light on the fact that the unique components of the CH_2_Cl_2_ fraction may contribute to the treatment or prevention of hypertension through their complex influence on the NO balance in the cardiovascular system.

Generally, it is well known that potassium chloride (KCl) opens voltage-dependent calcium channels by depolarizing the cell membrane of vascular smooth muscle, resulting in increased influx of extracellular Ca^2+^ [31]-[34]. Kim et al. [35] have shown that the contractile responses of vascular smooth muscle induced by CaCl_2_ and KCl may result most likely from the increased influx of extracellular Ca^2+^ through the voltage-dependent calcium channels (VDCCs). VDCCs are activated by depolarization of the plasma membrane when the extracellular K^+^ concentration is increased. In the present work, incubation with the CH_2_Cl_2_ fraction inhibited KCl concentration-dependent contractile response in rat aortic strips. This result is consistent with the effect of 17-β estradiol on a large elastic aorta as in previous reports [36],[37] and is also supported by another study [38]. These findings suggest that the CH_2_Cl_2_ fraction may have Ca^2+^ antagonistic properties and can inhibit extracellular Ca^2+^ influx through VDCCs, which are similar to those of 17-β estradiol or resveratrol. Generally, the mechanism of potassium-induced vasoconstriction has been shown to be through the calcium influx by the opening of the VDCCs [39],[40]. VDCC blockers such as nifedipine or verapamil have been reported to attenuate potassium-induced vasoconstriction [41],[42]. The contractile activity of vascular smooth muscle cells is mainly regulated by control over the cytoplasmic calcium concentration and both intracellular and extracellular calcium pools [42],[43]. Based on these findings, the present results that the CH_2_Cl_2_ fraction inhibited high K^+^-evoked contractile responses and that the inhibitory effect of the CH_2_Cl_2_ fraction on high K^+^-evoked contractile responses was enhanced, although their data are not shown here, indicate that the CH_2_Cl_2_ fraction may block the VDCCs in aortic smooth muscle cells.

In the present study, the finding that CH_2_Cl_2_ fraction-induced hypotension is suppressed by the pretreatment with an autonomic ganglionic blocker (chlorisondamine) and adrenergic α-blocker (phentolamine) suggests strongly that the CH_2_Cl_2_ fraction-induced hypotension may be mediated through the inhibition of sympathetic tone. The action site of the CH_2_Cl_2_ fraction seems to be the sympathetic ganglia a higher level because its hypotensive response is inhibited by prior treatment of chlorisondamine. Furthermore, in terms of the fact that intravenous CH_2_Cl_2_ fraction-evoked hypotension is significantly attenuated by adrenergic α-receptor blockade (phentolamine) and that the CH_2_Cl_2_ fraction inhibits greatly the pressor responses of norepinephrine, it is considered that the CH_2_Cl_2_ fraction causes the hypotensive action via the blockade of adrenergic α_1_-receptors. Among drugs which interfere with peripheral sympathetic function, adrenergic α-receptor blocking agents alone cause reversal of the epinephrine pressor response [44]. When epinephrine is administered to untreated animals, its α-agonist properties predominate, resulting in a rise in mean arterial pressure. However, in the presence of adrenergic α-receptor blockade, the peripheral β_2_-agonist properties of epinephrine predominate and a fall in arterial pressure or reversal of the pressor response is observed. In contrast, the pressor responses to norepinephrine are impaired by adrenergic α-receptor blockade but are not reversed [45] as this agent possesses little β_2_-agonist activity [46]. These previous facts support that CH_2_Cl_2_ fraction-induced depressor action is due to the blockade of adrenergic α-receptors in the periphery. In the present work, the CH_2_Cl_2_ fraction also inhibited the norepinephrine-induced pressor responses as well as PE-evoked contractile responses in aortic strips isolated from normotensive rats. These results suggest that the CH_2_Cl_2_ fraction may elicit the antagonistic activity of adrenergic α_1_-receptors.

Based on all these results, many studies strongly support the view that a polyphenol-rich diet, such as *Rubus coreanum* and red wine, could improve endothelial function and that the mechanisms of this beneficial effect found in the above-discussed *in vitro* studies (especially increased NO) might be involved *in vivo*, both in patients and in animals.

## Conclusion

In conclusion, the present study provides conclusive data showing for the first time that the CH_2_Cl_2_ fraction elicits endothelium- and NO-dependent vasorelaxation, which are due to the unique polyphenolic constituents of the CH_2_Cl_2_ fraction that may augment eNOS activity, and thus facilitates endothelial NO output, and suggesting that the CH_2_Cl_2_ fraction might be helpful in treating or alleviating cardiovascular diseases, such as hypertension and angina pectoris. The identification of the responsible constituents should help in the design of strategies to prevent or to improve cardiovascular diseases.

## Authors’ original submitted files for images

Below are the links to the authors’ original submitted files for images. Authors’ original file for figure 1 Authors’ original file for figure 2 Authors’ original file for figure 3 Authors’ original file for figure 4 Authors’ original file for figure 5 Authors’ original file for figure 6 Authors’ original file for figure 7 Authors’ original file for figure 8 Authors’ original file for figure 9

## Abbreviations

CH_2_Cl_2_ fraction: Methylene chloride (CH_2_Cl_2_) fraction
EtOAc fraction: Ethylacetate fraction
BuOH fraction: N-butanol fraction
PE: Phenylephrine
NO: Nitric oxide
PCRC: Polyphenol compounds isolated from *Rubus coreanum* MIQUEL
CA: Catecholamines
CHAPS: 3-[(3-cholamidopropyl) dimethylammonio]-1-propane sulfonate
DMSO: Dimethyl sulfoxide
L-NAME: N^ω^-nitro-L-arginine methyl ester hydrochloride
VDCCs: Voltage-dependent calcium channels
