# Supplementary figures and images for: Depressor action and vasorelaxation of methylene chloride fraction extracted from Rubus coreanum
Source: Clin Hypertens. 2014 Nov 25;20:6. doi: 10.1186/s40885-014-0006-1 (PMC4745144; doi:10.1186/s40885-014-0006-1)

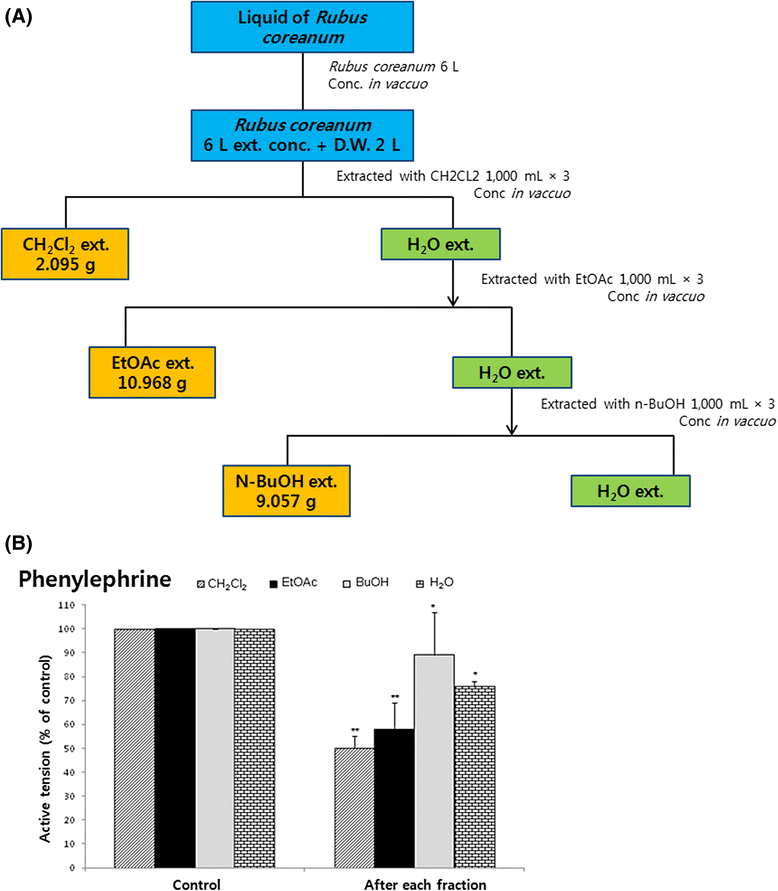

Supplement: Supplementary file 1 — Authors’ original file for figure 1 [file 40885_2014_6_MOESM1_ESM.gif]

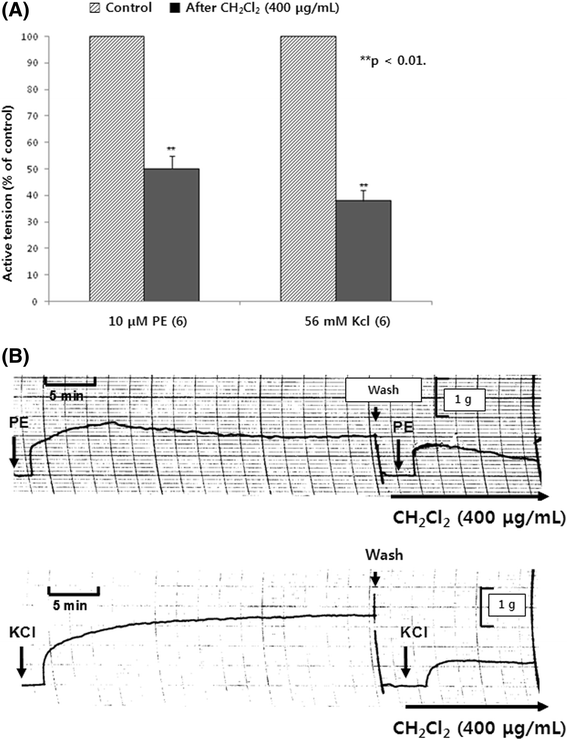

Supplement: Supplementary file 2 — Authors’ original file for figure 2 [file 40885_2014_6_MOESM2_ESM.gif]

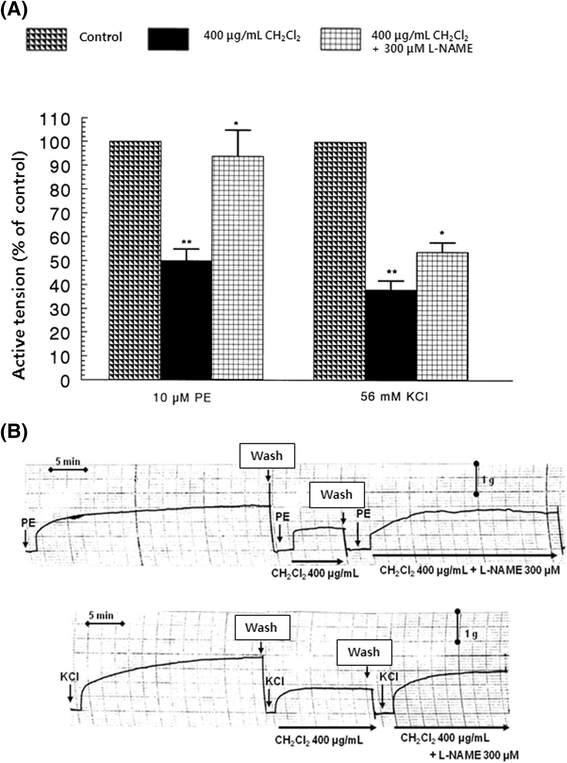

Supplement: Supplementary file 3 — Authors’ original file for figure 3 [file 40885_2014_6_MOESM3_ESM.gif]

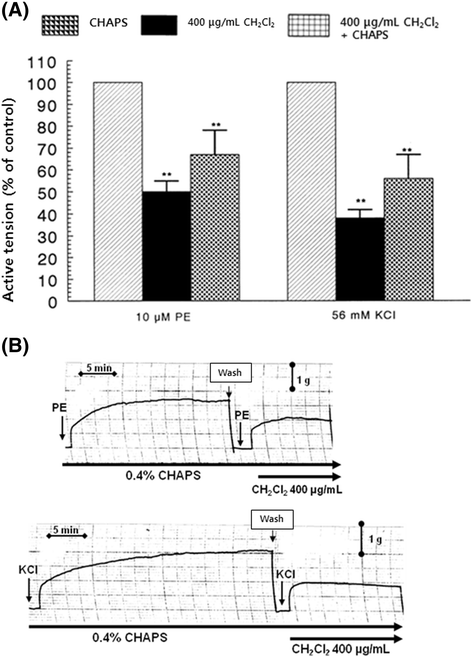

Supplement: Supplementary file 4 — Authors’ original file for figure 4 [file 40885_2014_6_MOESM4_ESM.gif]

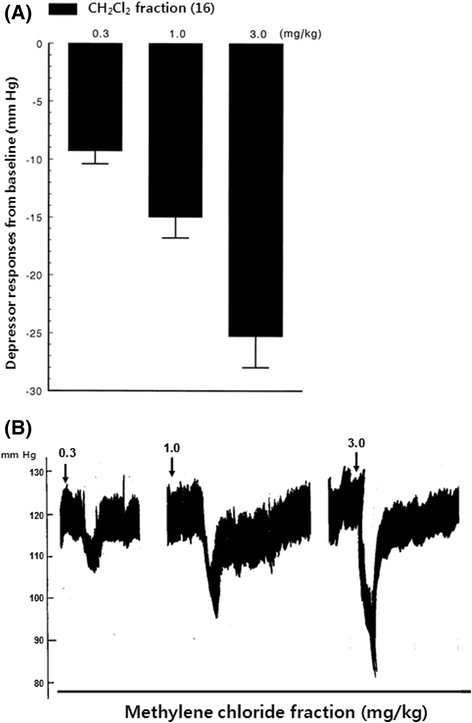

Supplement: Supplementary file 5 — Authors’ original file for figure 5 [file 40885_2014_6_MOESM5_ESM.gif]

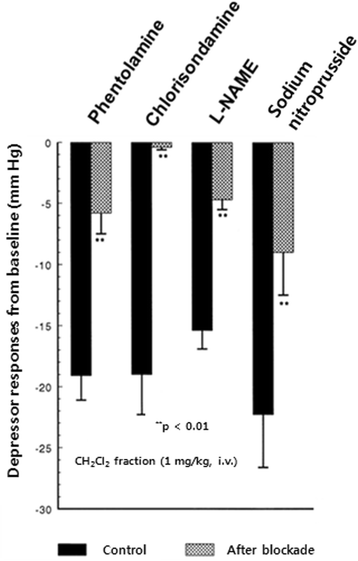

Supplement: Supplementary file 6 — Authors’ original file for figure 6 [file 40885_2014_6_MOESM6_ESM.gif]

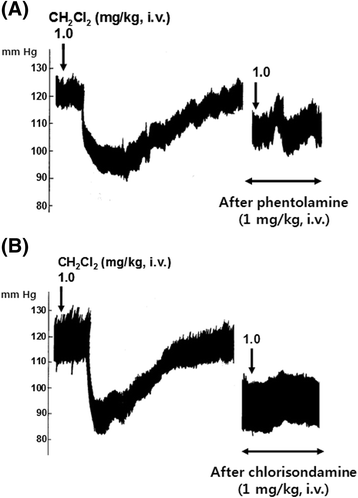

Supplement: Supplementary file 7 — Authors’ original file for figure 7 [file 40885_2014_6_MOESM7_ESM.gif]

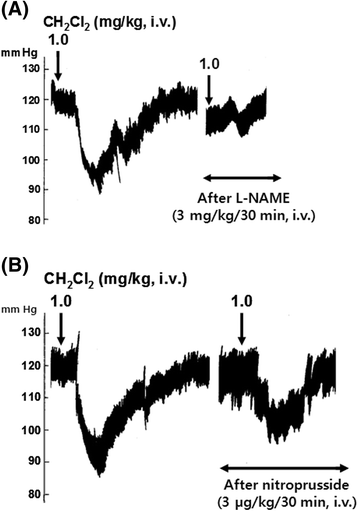

Supplement: Supplementary file 8 — Authors’ original file for figure 8 [file 40885_2014_6_MOESM8_ESM.gif]

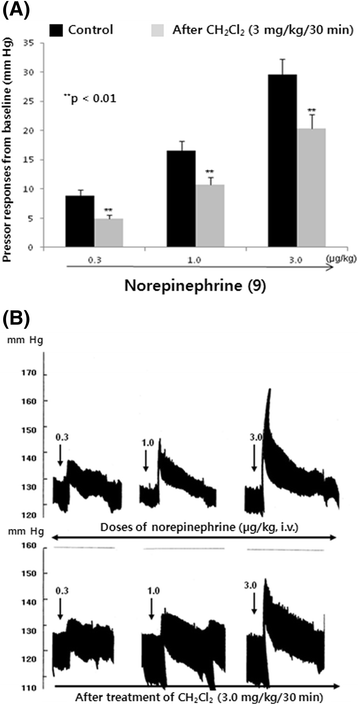

Supplement: Supplementary file 9 — Authors’ original file for figure 9 [file 40885_2014_6_MOESM9_ESM.gif]
